# Multi-topic assignment for exploratory navigation of consumer health information in NetWellness using formal concept analysis

**DOI:** 10.1186/1472-6947-14-63

**Published:** 2014-08-03

**Authors:** Licong Cui, Rong Xu, Zhihui Luo, Susan Wentz, Kyle Scarberry, Guo-Qiang Zhang

**Affiliations:** 1 Department of Electrical Engineering and Computer Science, Case Western Reserve University, Cleveland OH 44106, USA; 2 Division of Medical Informatics, School of Medicine, Case Western Reserve University, Cleveland OH 44106, USA; 3 School of Medicine, Case Western Reserve University, Cleveland OH 44106, USA; 4 University Hospitals Case Medical Center, Cleveland OH 44106, USA

**Keywords:** Consumer health information, Multi-topic assignment, Formal concept analysis, Navigational exploration

## Abstract

**Background:**

Finding quality consumer health information online can effectively bring important public health benefits to the general population. It can empower people with timely and current knowledge for managing their health and promoting wellbeing. Despite a popular belief that search engines such as Google can solve all information access problems, recent studies show that using search engines and simple search terms is not sufficient. Our objective is to provide an approach to organizing consumer health information for navigational exploration, complementing keyword-based direct search. Multi-topic assignment to health information, such as online questions, is a fundamental step for navigational exploration.

**Methods:**

We introduce a new multi-topic assignment method combining semantic annotation using UMLS concepts (CUIs) and Formal Concept Analysis (FCA). Each question was tagged with CUIs identified by MetaMap. The CUIs were filtered with term-frequency and a new term-strength index to construct a CUI-question context. The CUI-question context and a topic-subject context were used for multi-topic assignment, resulting in a topic-question context. The topic-question context was then directly used for constructing a prototype navigational exploration interface.

**Results:**

Experimental evaluation was performed on the task of automatic multi-topic assignment of 99 predefined topics for about 60,000 consumer health questions from NetWellness. Using example-based metrics, suitable for multi-topic assignment problems, our method achieved a precision of 0.849, recall of 0.774, and *F*_1_ measure of 0.782, using a reference standard of 278 questions with manually assigned topics. Compared to NetWellness’ original topic assignment, a 36.5% increase in recall is achieved with virtually no sacrifice in precision.

**Conclusion:**

Enhancing the recall of multi-topic assignment without sacrificing precision is a prerequisite for achieving the benefits of navigational exploration. Our new multi-topic assignment method, combining term-strength, FCA, and information retrieval techniques, significantly improved recall and performed well according to example-based metrics.

## Background

The Internet provides an important source of consumer health information to patients, caregivers, families, and laypersons. The proliferation of online health information from government agencies, non-profit organizations, for-profit companies, and chatting and social networking sites presents myriad of challenges for information access. For example, a study reported in JAMA [1] found that accessing health information using search engines and simple search terms is not sufficient. Even though the accuracy of information found on selected common topics is good among the top 14 selected sites, coverage is poor and inconsistent. In some cases, more than 10 steps of web-links did not lead to relevant information within the search results obtained.

One approach to addressing such challenges is to complement direct search with mechanisms for navigational exploration, one of the two basic modes for information access [2-4]: 

1. **Direct search**, where a user, knowing what to look for, comes with specific pieces of information about a disease, procedure, or medication, or other related description and tries to retrieve a corresponding set of responses. For example, direct search is most effective for looking up the side effects of medications such as Warfarin, using the drug name as a key search string.

2. **Navigational exploration**, where the goal is to explore and see “what is there.” For example, finding answers to questions such as “Other than prescribed medications, what are alternatives that may help with depression” involve more exploring than searching. In this mode, the consumer may not be able to easily and effectively formulate a descriptive direct search, and must rely on navigational menus or “facets” [4-6] to browse and explore the content.

In most cases, direct search may be accompanied by navigational exploration to help the user “finding needles in a haystack:” the volume of search results can be overwhelmingly large and needs to be further structured to allow relevant information to be located. For example, the same JAMA study [1] reported 3,735 links in the first page of search results from the 14 selected sites. Among 389 sampled relevant links, about a quarter did not lead to a content page within 10 clicks. This demonstrates that a mechanism for navigational exploration, complementing direct search, should be helpful. In related work, Mu et al. [7] presented a facet-view information navigation interface called SimMed complementing lookup search for effectively retrieving medical literatures in a subset of MEDLINE. More recently, Cui et al. [8] introduced a conjunctive exploratory navigation interface called CENI for supporting effective retrieval of consumer health questions.

**Figure 1:**
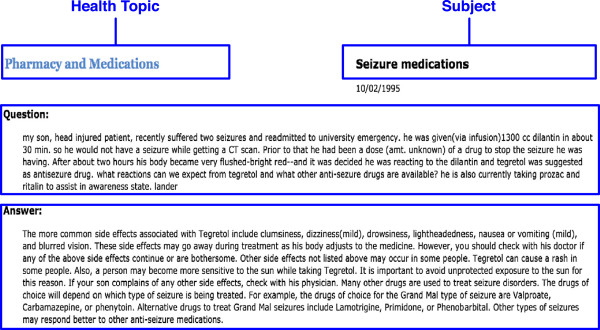
**A sample consumer question in NetWellness.** Each question has four major components: Health Topic, Subject, Question, and Answer.

Consumer experience in navigational exploration mode depends on *Information Organization*, a topic centered around structures (e.g. nested folder or menu hierarchies) with which to organize a collection of contents to facilitate browsing and exploration. For example, Community Question Answering (CQA) services on the web enable users to ask and answer questions. Such services for consumer health include WebMD Answers (http://answers.webmd.com/) and NetWellness (http://netwellness.org/). Questions in CQA services are often organized into categories or topics to facilitate browsing, exploring and searching questions and answers.However, a common limitation of these organizational structures is that each question is assigned a single topic among a collection of topics, even though multiple topics are often relevant. For example, Figure 1 shows a health question in NetWellness, which was assigned a single topic “Pharmacy and Medications,” but it is also related to “Epilepsy.” Allowing for a single question being assigned multiple relevant topics (if applicable) enables consumers to reach it through multiple pathways, thus improving the retrieval recall in the navigational exploration mode.

The categorization of a question into multiple topics (a.k.a. **multi-topic assignment**) is a multi-label classification problem, a complex task where each item (question) can be associated with one or more labels (topics).

Existing machine learning methods for multi-label classification can be grouped into two main categories: *problem transformation*, which converts the multi-label classification problem into multiple single-label classification problems, and *algorithm adaptation*, which extends specific learning algorithms for single-label classification problems to handle multi-label data directly (see [9,10] for the detailed methods). Several existing work involves multi-label classification in clinical research. A clinical coding challenge [11] organized by the Cincinnati Children’s Hospital Medical Center in 2007 focused on the assignment of ICD-9-CM codes to radiology reports. In [12], supervised binary classifiers were developed to assign 12 predefined general topics (e.g. etiology, procedure, and diagnosis) to clinical questions.

In this paper we introduce a new multi-topic assignment method combining Formal Concept Analysis (FCA [13]) and semantic annotation using Unified Medical Language System (UMLS) [14]. Each health question in NetWellness was tagged with UMLS Concept Unique Identifiers (CUIs) identified by MetaMap. The CUIs were filtered with term-frequency and a new term-strength index to construct a CUI-question context. The CUI-question context and a topic-subject context were used for multi-topic assignment, resulting in a topic-question context. The topic-question context was then directly used for constructing a prototype navigational exploration interface called Concept-guided Automatic Organization of Consumer Health information (iCOACH).

## Methods

Formal Concept Analysis (FCA)[13] is a lattice-based method for extracting higher-level organizational information from lower-level classification of objects according to their attributes. FCA builds from a formal context (or context), (*O*,*A*,*R*), with *O* a collection of objects (e.g. questions), *A* a collection of attributes (e.g. topics), and *R* a binary relation from *O* to *A*. *R* is specified by a table, where a “ ×” entry indicates the relation between corresponding object (row) and the corresponding attribute (column). FCA clusters objects into a concept hierarchy (called a concept lattice), suitable for visualization and quantitative analysis with considerable organizational power. Each logical cluster is called a formal concept, representing a basic unit of information by harmonizing subsets of objects (the **extent**) and their associated attributes (the **intent**) using a closure operation.

**Figure 2:**
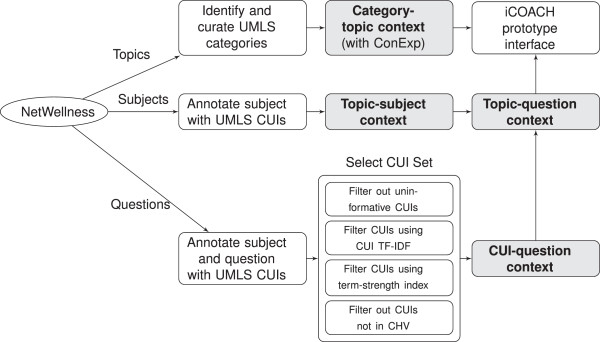
**The workflow for organizing NetWellness consumer health questions for navigational exploration.** Four formal contexts in FCA are the nodes shaded in grey. The category-topic context drives the dynamic, on-the-fly identification of topic groups according to selected categories in the iCOACH prototype interface; The topic-subject context and CUI-question context are used to classify a question with multiple topics, resulting in the topic-question context, which drives the dynamic, on-the-fly identification of question groups according to selected topics in the iCOACH prototype interface. The process to create the category-topic context involves manual curation using ConExp [15], all other processes are done automatically.

We use FCA to categorize questions into multiple topics for content organization and to drive a dynamically navigational exploration interface for content-group generation. Each use of FCA involves the creation of a formal context and the dynamic generation of the corresponding formal concept (i.e. its intent and extent) based on a selected subset of attributes.Our approach involves the identification of attributes, objects, and construction of formal contexts (or just contexts), and the integration and coordination among the contexts (see Figure 2). Four formal contexts were developed: 

1. *Category-topic context*. Health categories were adapted from a subset of UMLS’ semantic types to organize NetWellness topics. The category-topic context classified topics to relevant categories to indicate if a topic “is a” or “typically involves” the corresponding category. It was used to dynamically create a list of topics (extent) in the intersection of a selected set of categories (intent).

2. *Topic-subject context*. This context took subjects and topics as objects and attributes respectively. The subject of each question was annotated with UMLS CUIs, representing a set of semantic concepts involved in the subject. It provided a way to assign multiple topics to a given question.

3. *CUI-question context*. Each question and its subject were annotated with UMLS CUIs, representing a set of semantic concepts involved in the question. The CUIs were filtered with term-frequency and a new term-strength index to construct the CUI-question context. This context was used as another way to assign multiple topics to questions.

4. *Topic-question context*. This context categorized questions into multiple topics using the topic-subject context and CUI-question context. It was used to dynamically create a list of questions (extent) in the intersection of a selected set of topics (intent).

### Category-topic context

Health categories (or categories) are the highest level of labels used to group health topics. We used UMLS semantic types as the candidate pool for creating categories that are meaningful for consumers. To be effective, we only used content-specific semantic types for this purpose, and relied on MetaMap [16] to identify a subset of NetWellness-relevant semantic types. This subset was further regrouped and sometimes relabeled with the help of two NetWellness experts, to narrow down to a dozen of manageable top-level categories. These categories are “Activity and Behavior,” “Anatomy and Body System,” “Disease, Syndrome and Disorder,” “Drugs, Medication and Substance,” “Environmental and Risk Factors,” “Health and Wellbeing,” “Inheritance, Genetics and Genomics,” “Medical Device,” “Population and Subgroups,” “Prevention and Screening,” “Procedure and Process,” and “Symptom or Sign.” The curated 12 categories and 99 topics were used as the attributes and objects for FCA respectively. The corresponding formal context was manually created and verified by the NetWellness experts. ConExp [15] is a Java-based open-source FCA tool for editing formal context, building and visualizing concept lattices from a formal context, and performing attribute exploration. ConExp was used to assist the editing and validation process of constructing the category-topic context.

### Topic-subject context

The topic-subject context serves as a bridge between topics and questions. Both topics and subjects are represented as CUI-sets. We manually curated a list *L* of 140 key topic CUIs, and each CUI can directly determine a topic. For each such topic, a set of synonyms and descendants of its CUIs in *L* was automatically collected using UMLS’ semantic relations to construct the topic CUI-set, and the topic served as an attribute for FCA. Table 1 lists some topics and their corresponding key topic CUIs. Table 2 shows the CUI-set for the topic “Pregnancy” including 21 CUIs.

**Table 1:** Examples of topics and their corresponding key topic CUIs

**Topic**	**Key topic CUIs**
Anesthesia	C0278134 (Anaesthesia),
	C0002915 (General Anesthesia),
	C0002903 (Anesthesia procedure)
Asthma	C0004096 (Asthma),
	C2984299 (Asthma Pathway)
Breast Feeding	C0006147 (Breast Feeding),
	C1623040 (Breastfeeding (mother))
Pain Management	C0002766 (Pain management),
	C0030193 (Pain)
Pharmacy and	C0031322 (Pharmacy facility),
Medications	C0013227 (Pharmaceutical Preparations),
	C0802604 (Medications)
Pregnancy	C0032961 (Pregnancy), C0553641(Pregnant),
	C0549206 (Patient currently pregnant)

**Table 2:** The CUI-set for the topic “Pregnancy” (21 CUIs)

C0032961: Pregnancy	C0553641: Pregnant
C0585066: Mother currently	C0041747: Unplanned pregnancy
breast-feeding	
C0232989: Normal pregnancy	C0404831: Multigravida
C0549206: Patient currently	C0032995: Unwanted pregnancy
pregnant	
C1291689: Number of	C0242786: High-Risk Pregnancy
pregnancies, currently pregnant	
C0425984: Pregnant - on history	C0425983: Pregnant - on abdominal
	palpation
C0425986: Pregnant - blood test	C0425985: Pregnant - V.E. confirms
confirms	
C0149973: Intrauterine pregnancy	C0425987: Pregnant - urine test
	confirms
C0232993: Extrachorial pregnancy	C0232992: Extra-amniotic pregnancy
C0232990: Precocious pregnancy	C0404842: Surrogate pregnancy
	C2586154: Intends to continue
	pregnancy

The subject of a question is usually short but more likely to convey important information. Each subject had a CUI-set assigned by MetaMap, and served as an object for FCA. Table 3 lists some subjects and their annotated CUIs.

**Table 3:** Examples of subjects and their annotated CUIs

**Subject**	**Annotated CUIs**
Safety of general	C1705187 (Safety),
anesthesia	C0002915 (General Anesthesia)
Breast Feeding and	C1623040 (Breast feeding),
Asthma Medications	C0004096 (Asthma),
	C0013227 (Pharmaceutical Preparations)
Asthma and pregnancy	C0004096 (Asthma), C0032961 (Pregnancy)
Hemorrhoids accompanied	C0019112 (Hemorrhoids),
by abdominal pain	C0000737 (Abdominal Pain)
Breastfeeding and getting	C0006147 (Breast Feeding),
pregnant	C0549206 (Patient currently pregnant)
Pregnancy while on	C0032961 (Pregnancy),
TB Medications	C0802604 (Medications)

The corresponding formal context was automatically generated using subject CUI-sets and topic CUI-sets. In this context, a “ ×” entry corresponding to an object (subject) and an attribute (topic) indicates that the intersection of the subject CUI-set and the topic CUI-set is not empty.

Since each key topic CUI in *L* can determine a topic, its synonyms and descendant can also determine the topic. Therefore, if a subject is annotated with at least one CUI in a topic CUI-set (that is, the intersection of the subject CUI-set and the topic CUI-set is not empty), then a “ ×” entry is added to the topic-subject context indicating that the subject relates to the topic. Table 4 shows the topic-subject context determined by the topics in Table 1 and the subjects in Table 3.

**Table 4:** **The topic-subject context determined by the topics in Table **1** and the subjects in Table **3

	***R***	**Topics (attributes)**					
		**Anesthesia**	**Asthma**	**Breast feeding**	**Pain management**	**Pharmacy and**	**Pregnancy**
						**medications**	
**Subjects (objects)**	Safety of general anesthesia	×					
	Breast feeding and asthma medications		×	×		×	
	Asthma and pregnancy		×				×
	Hemorrhoids accompanied by abdominal pain				×		
	Breastfeeding and getting pregnant			×			×
	Pregnancy while on TB Medications					×	×

### CUI-question context

The CUI-question context serves as another bridge between topics and questions. Terms in each question and its subject were annotated with UMLS CUIs and semantic types using MetaMap. This obtained 32042 distinct CUIs for all questions as well as their subjects. Since there is a large number of CUIs involved and not all of them are relevant to consumer heath, we used the following steps to select a subset of most relevant CUIs:

*Step 1: Filtering out uninformative CUIs by their semantic types.* We manually excluded a collection of semantic types that are not relevant to our task of assigning topics to questions (e.g., “Quantitative Concept,” “Intellectual Product,” “Geographic Area,” and “Organization”). CUIs with excluded semantic types were filtered out for each question. A total of 23802 distinct CUIs were left after this step.

*Step 2: Filtering CUIs using CUI TF-IDF.* The CUI-set for each question was filtered using TF-IDF [17]. CUI term-frequency (TF) was calculated as the number of occurrences of a CUI in a question and its subject (key CUIs are usually mentioned in the subject and repeated one or several times in the question), normalized by the number of all CUI occurrences in that question and its subject. The inverse document frequency (IDF) was used to measure the importance of a CUI in the corpus of all questions; it is the logarithm of the quotient of the number of all questions and the number of questions containing the CUI. The TF-IDF weight, TF ×IDF, was used to determine the importance of a CUI for a question. For each question, the top five ranked CUIs were selected. A total of 21212 distinct CUIs were left after performing CUI TF-IDF.

*Step 3: Filtering CUIs using a term-strength index.* To automatically find associated questions for a given question and to assign multiple topics to questions, co-occurrences of CUIs were taken into account. We defined a term-strength index for CUIs as follows: Given a CUI *C*, we use {(*C*_*i*_,*N*_*i*_)|*i*=1,2,…,*k*} to denote *C*’s co-occurring CUI list among all the questions, where *C*_*i*_ is C’s co-occurring CUI and *N*_*i*_ represents the number of questions containing both *C*_*i*_ and *C*, and *k* is the total number of *C*’s co-occurring CUIs. A CUI *C* has term-strength index *n* if *n* of *C*’s co-occurring CUIs have at least *n* common questions each, and the other (*k*−*n*) co-occurring CUIs at most *n* common questions each. That is,

$$n=\max\left\{j\;\;\left|\;\;\left|\left\{N_{i}|N_{i}\geq j\right\}\right)\right|\geq j,j\leq k\right\}$$

where |{*N*_*i*_∣*N*_*i*_≥*j*}| is the size of the set {*N*_*i*_∣*N*_*i*_≥*j*}. The top 20 CUIs ranked by the term-strength index are shown in Table 5. The CUI-set for each question was then reformulated by filtering out CUIs whose term-strength indexes are less than 2. This step resulted in a total of 8208 distinct CUIs.

**Table 5:** Top 20 CUIs ranked by our term-strength index

**CUI**	**Concept name**	**Term-strength index**
C0549206	Patient currently pregnant	23
C0015392	Eye	20
C0030193	Pain	19
C0019080	Hemorrhage	19
C0040408	Tongue	19
C0040426	Tooth structure	18
C0021270	Infant	17
C0015127	Etiology aspects	16
C0013443	Ear structure	16
C0577559	Mass of body structure	15
C0031354	Pharyngeal structure	15
C0032961	Pregnancy	15
C0009253	Coitus	14
C0008059	Child	14
C0013227	Pharmaceutical Preparations	14
C0013470	Eating	14
C0024109	Lung	14
C0038999	Swelling	13
C0543467	Operative Surgical Procedures	13
C0022646	Kidney	13

*Step 4: Filtering out CUIs not in the Consumer Health Vocabulary (CHV)*[18]. Removing CUIs that are not in CHV obtained 7127 distinct CUIs.

*Step 5: Creating CUI-question context.* The CUI-question context was automatically generated by the questions and their relevant CUIs left after the previous four steps. Table 6 shows a subcontext of this context, where the attributes are three CUIs: C0232908 (concept name: “conceived”), C0025874 (concept name: “Metrorrhagia”), and C0439531(concept name: “period”), and the objects are eight questions labeled as Q1, Q2, Q3, Q4, Q5, Q6, Q7, and Q8.

**Table 6:** A subcontext of the CUI-question context

		**CUIs**		
		**C0232908**	**C0025874**	**C0439531**
		**(conceived)**	**(Metrorrhagia)**	**(Period)**
**Questions**	Q1		×	×
	Q2	×	×	
	Q3	×	×	
	Q4	×	×	×
	Q5		×	×
	Q6		×	×
	Q7	×	×	×
	Q8		×	×

The terms in parentheses are the concept names of the CUIs. The original NetWellness topics assigned for each question are: Q1: “Gynecology,” Q2: “Pregnancy,” Q3: “Pregnancy,” Q4: “Pregnancy,” Q5: “Gynecology,” Q6: “Infertility,” Q7: “Women’s Health,” and Q8: “Gynecology.” The final topic assignments using FCA are Q1: “Gynecology,” Q2: “Pregnancy,” Q3: “Pregnancy,” Q4: {“Pregnancy,” “Gynecology”}, Q5: “Gynecology,” Q6: {“Infertility,” “Gynecology”}, Q7: {“Women’s Health,” “Gynecology,” “Pregnancy”}, and Q8: “Gynecology”.

### Topic-question context: a question may be assigned multiple topics

The construction of topic-question context relies on the tagging of a question by multiple topics. We developed two methods of assigning topics to questions using (1) topic-subject context, and (2) CUI-question context.

#### Categorizing questions using the topic-subject context

The CUI-sets for most topics were constructed by first identifying CUIs directly associated with the topics, and then adding additional CUIs through the UMLS knowledge source. A question was categorized to a topic if the intersection of its subject CUI-set and the topic CUI-set is not empty. This has the effect of putting significant weight on a question’s subject content, which is consistent with our intuition. Since the pre-defined NetWellness topics are not incomparable, an associated set of rules reflecting the hierarchical relationship between topics was also used in the topic assignment. Assigning multiple topics to questions results in a comprehensive topic-question context, thus allowing consumers to quickly narrow down to relevant questions in iCOACH’s conjunctive organization framework while not missing those questions that are relevant to a topic.

#### Categorizing questions using the CUI-question context

For each question in the CUI-question context, its CUI-set determines a formal concept computed using the Colibri-Java library [19]. This formal concept is a set of questions (extent) sharing the CUIs (intent) in the CUI-set of the given question. Among the questions in the extent of the formal concept, those having exactly the same CUI-set as the given question as well as their originally assigned NetWellness topics are utilized to assigned topics to questions by leveraging a voting scheme. For instance, in the context shown in Table 6, originally NetWellness assigned topic for each question is Q1: “Gynecology,” Q2: “Pregnancy,” Q3: “Pregnancy,” Q4: “Pregnancy,” Q5: “Gynecology,” Q6: “Infertility,” Q7: “Women’s Health,” and Q8: “Gynecology.”

Q1’s CUI-set {C0025874 (“Metrorrhagia”), C0439531 (“period”)} determines a set of associated questions {Q1, Q4, Q5, Q6, Q7, Q8}. Among these associated questions, Q1, Q5, Q6, and Q8 have the same CUI-set as Q1, and their originally NetWellness assigned topics (Q1: “Gynecology,” Q5: “Gynecology,” Q6: “Infertility,” Q8: “Gynecology”) are considered as the potential candidate topics for the associated questions. The best candidate topics are decided by a voting scheme, where each question contributes to a vote for its original topic (“Gynecology” has 3 votes, and “Infertility” has 1 vote). The topics excluding those with 1 vote are considered as the best candidate topics for the associated question group. Therefore, Q1, Q4, Q5, Q6, Q7, and Q8 are assigned the topic “Gynecology,” where Q4, Q6, and Q7 receive a new topic “Gynecology,” while Q1, Q5, and Q8 receive no new topics. As a result, the topic assignment determined by Q1’s CUI-set is Q1: “Gynecology,” Q2: “Pregnancy,” Q3: “Pregnancy,” Q4: {“Pregnancy,” “Gynecology”}, Q5: “Gynecology,” Q6: {“Infertility,” “Gynecology”}, Q7: {“Women’s Health,” “Gynecology”}, and Q8: “Gynecology.”

Q2’s CUI-Set {C0232908 (“conceived”), C0025874 (“Metrorrhagia”)} determines a set of associated questions {Q2, Q3, Q4, Q7}, among which Q2 and Q3 have the same CUI-set as Q2, and their originally NetWellness assigned topics (Q2: “Pregnancy,” Q3: “Pregnancy”) are considered as the potential candidate topics for the associated questions. Since “Pregnancy” receives 2 votes, it is considered as the best candidate topic for the associated questions. Therefore, Q7 receives a new topic “Pregnancy,” while Q2, Q3, and Q4 receive no new topics. As a result, the topic assignment determined by Q2’s CUI-set is Q1: “Gynecology,” Q2: “Pregnancy,” Q3: “Pregnancy,” Q4: {“Pregnancy,” “Gynecology” }, Q5: “Gynecology,” Q6: {“Infertility,” “Gynecology”}, Q7: {“Women’s Health,” “Gynecology,” “Pregnancy”}, and Q8: “Gynecology.” Performing the similar process for the remaining questions and the final topic assignment keeps the same as the topic assignment determined by Q2’s CUI-set.

### Evaluation metrics

Example-based measures for multi-label classification problems were used as the evaluation metrics [9,10]. Let *L* be a set of labels, and *D* be a multi-label evaluation data set consisting of *m* multi-label examples (*x*_*i*_,*Y*_*i*_), where *i*∈{1,…,*m*} and *Y*_*i*_⊆*L*. Let *Z*_*i*_ be the set of predicted labels for *x*_*i*_. The example-based precision (*P*), recall (*R*) and *F*_1_ measure (*F*_1_) are defined as follows:

(1) $$P=\frac{1}{m}\overset{m}{\underset{i=1}{\sum }}\frac{\left|Y_{i}\cap Z_{i}\right|}{\left|Z_{i}\right|},$$

(2) $$R=\frac{1}{m}\overset{m}{\underset{i=1}{\sum }}\frac{\left|Y_{i}\cap Z_{i}\right|}{\left|Y_{i}\right|},\text{and}$$

(3) $$F_{1}=\frac{1}{m}\overset{m}{\underset{i=1}{\sum }}\frac{2|Y_{i}\cap Z_{i}|}{\left|Z_{i}|+|Y_{i}\right|}.$$

Example-based measures described above have been designed to avoid undue influence of a few questions with an unusually large number of labels. For a multi-label classification problem, the traditional measures could potentially allow the performance on a few such questions to dominate the value of the metric. See Appendix for an example illustrating the distinctions between example-based measures and traditional measures.

### Reference standard development

To create a reference standard, 300 questions were randomly selected using stratified sampling from NetWellness’ pool of over 60,000 questions. We developed a web-based interface for annotators to tag questions with topics. Three annotators (A1, A2, A3) generated the reference standard in two iterations. In the initial iteration, the annotators assigned topics to questions independently. The standard kappa statistic cannot be used to measure agreement between annotators in this work since it assumes each item is assigned a unique label [20], even though the candidate labels can be several. For our task, each question can be assigned one or multiple topics. We adapted the idea of computing inter-annotator agreement (IAA) using *F*_1_ measure [21], where one annotator’s annotations were used as the reference standard to calculate the *F*_1_ measure of the other annotator. We computed example-based *F*_1_ measures for pairs of annotators: (A1, A2)-0.819, (A1, A3)-0.828, (A2, A3)-0.755. The average example-based *F*_1_ measure among all pairs of annotators is 0.801, which showed reasonably good inter-annotator agreement. In the second iteration, the annotators discussed and resolved the disagreements and finalized the reference standard consisting of 278 questions. 22 questions were excluded since they were not informative for topic assignment, or there were no available topics to assign. In total, 497 topics were assigned to these 278 questions.

#### Ethics statement

This study involved no human subjects, and qualified as an exempt research activity under the Code of Federal Regulations [38 CFR 16.101(b) Section 3, Category 2].

## Results

### Summary of contexts

The category-topic context consisted of 12 categories and 99 topics. The concept lattice of this context consists of 71 concept nodes (Additional file 1: Figures S2). The CUI-question context involved 7127 CUIs and 54184 questions. The topic-subject context consisted of 91 topics and 54787 subjects. Three methods were used to construct the topic and question context: (1) Categorizing questions using the CUI-question context gave a topic-question context containing 80781 “ ×” entries; (2) Using the topic-subject context resulted in 82800 “ ×” entries; and (3) Combining (1) and (2) obtained 92034 “ ×” entries.

### Evaluation

For performance evaluation, we focused on improving the key metric of recall, representing the most important area for improvement that affects the experience of a user’s navigational exploration. In the reference standard, 278 questions have 497 topics assigned in total. The performance of the two methods categorizing questions and their combination were compared with that of NetWellness’ originally manual, single-topic assignment against the reference standard.

Table 7 shows the total numbers of correctly assigned, wrongly assigned, and missing topics for the original NetWellness’ and our multi-topic assignment methods against the reference standard. Combination of using topic-subject context and using CUI-question context produced the largest number of correct topics (364) and smallest number of missing topics (133). It also achieved the best result: an example-based precision of 0.849, recall of 0.774, and *F*_1_ measure of 0.782. Compared to NetWellness’ manual, single-topic assignment, a 36.5% increase in recall (from 0.567 to 0.774) was achieved with no sacrifice of precision.

**Table 7:** **The example-based precision, recall, and *F***_**1**_** measures for original NetWellness’ assignments and our multi-topic assignments**

**Method**	**Correctly**	**Wrongly**	**Missing**	**Precision**	**Recall**	***F***_**1**_
	**assigned topics**	**assigned topics**	**topics**			
Original NetWellness	234	44	263	0.842	0.567	0.649
Using topic-subject context	346	56	151	0.873	0.746	0.781
Using CUI-question context	318	69	179	0.835	0.7	0.731
Combination of the above two	364	77	133	0.849	0.774	0.782

These numbers are reported for the 278 questions with 497 topics in the reference standard.

Table 8 shows the performance of our combination method for the top 10 topics ranked by the number of questions appearing in the reference standard, compared to original NetWellness’ topic assignment (questions in the reference standard involved 77 topics according to the original NetWellness’ assignment). For our combined approach, “Pregnancy” achieved the best example-based recall (0.949) and *F*_1_ measure (0.933), and “Diet and Nutrition” had the lowest example-based *F*_1_ measure (0.625).

**Table 8:** **The example-based precision, recall, and** ***F***_**1**_** measure for the top 10 topics**

**Topic**	**RF**	**Method**	**TC**	**TW**	**TM**	**Precision**	**Recall**	***F***_**1**_
Gynecology (17)	39	NetWellness	12	5	27	0.706	0.328	0.445
		FCA	32	7	7	0.853	0.853	0.841
Pharmacy and Medications (17)	27	NetWellness	15	2	12	0.882	0.667	0.735
		FCA	17	3	10	0.853	0.716	0.753
ENT Disorder (15)	19	NetWellness	13	2	6	0.867	0.767	0.8
		FCA	14	3	5	0.867	0.8	0.822
Pregnancy (13)	32	NetWellness	13	0	19	1.0	0.423	0.59
		FCA	30	3	2	0.942	0.949	0.933
Children’s Health (12)	19	NetWellness	12	0	7	1.0	0.764	0.833
		FCA	12	3	7	0.875	0.764	0.764
Eye and Vision Care (11)	17	NetWellness	11	0	6	1.0	0.727	0.818
		FCA	13	0	4	1.0	0.818	0.879
Myasthenia Gravis (11)	21	NetWellness	9	2	12	0.818	0.561	0.636
		FCA	15	2	6	0.864	0.765	0.8
Women’s Health (10)	18	NetWellness	9	1	9	0.9	0.533	0.65
		FCA	12	3	6	0.8	0.683	0.7
Diet and Nutrition (8)	11	NetWellness	6	2	5	0.75	0.563	0.625
		FCA	6	2	5	0.75	0.563	0.625
Urinary and Genital Disorders (8)	13	NetWellness	7	1	6	0.875	0.667	0.729
		FCA	8	2	5	0.917	0.729	0.779

The top 10 topics are ranked by the number of questions (displayed in the parentheses) in the reference standard categorized by the corresponding topic in the original NetWellness assignment. The performance of the original NetWellness assignment and our combined method (see Table 7) denoted as FCA are displayed. RF: Total number of topics assigned in reference standard; TC: Total number of correctly assigned topics; TW: Total number of wrongly assigned topics; TM: Total number of missing topics.

### iCOACH prototype interface

**Figure 3:**
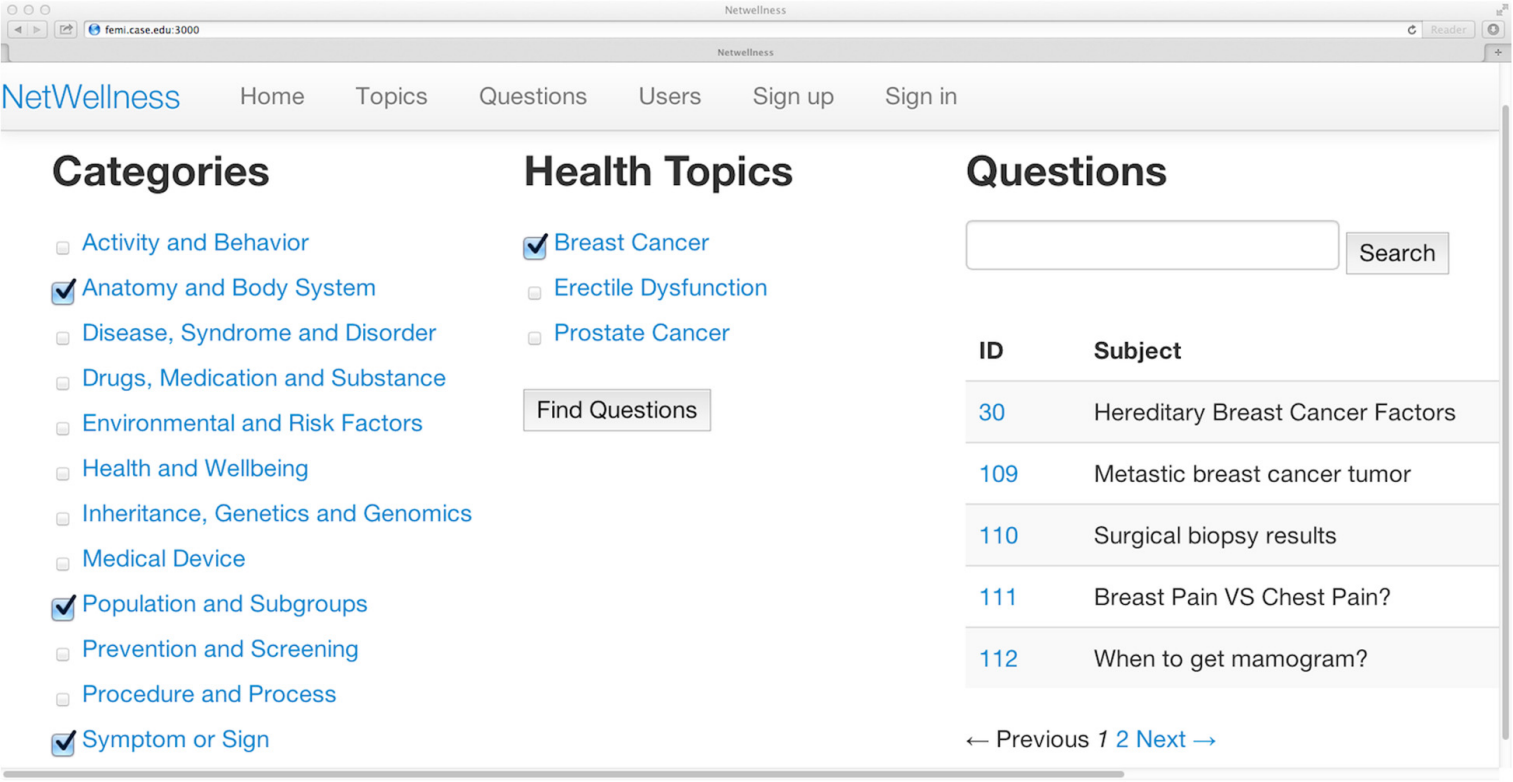
**A screenshot of the conjunctive navigation interface.** The category-topic context drives the on-the-fly allocation of a set of topics related to a select set of categories (leftmost column), with a total of 71 possible variations corresponding to the 71 concept nodes. Selection of multiple categories, such as “Anatomy and Body System,” “Population and Subgroups,” and “Symptom or Sign” (leftmost column) immediately guides the consumer to relevant topics (middle column) lying at the intersection of all the categories (conjunctively), rather than those belonging to the union of all the categories (disjunctively). The topic-question context drives the dynamic generation of a set of questions (rightmost column) relating to a selected set of topics.

**Figure 4:**
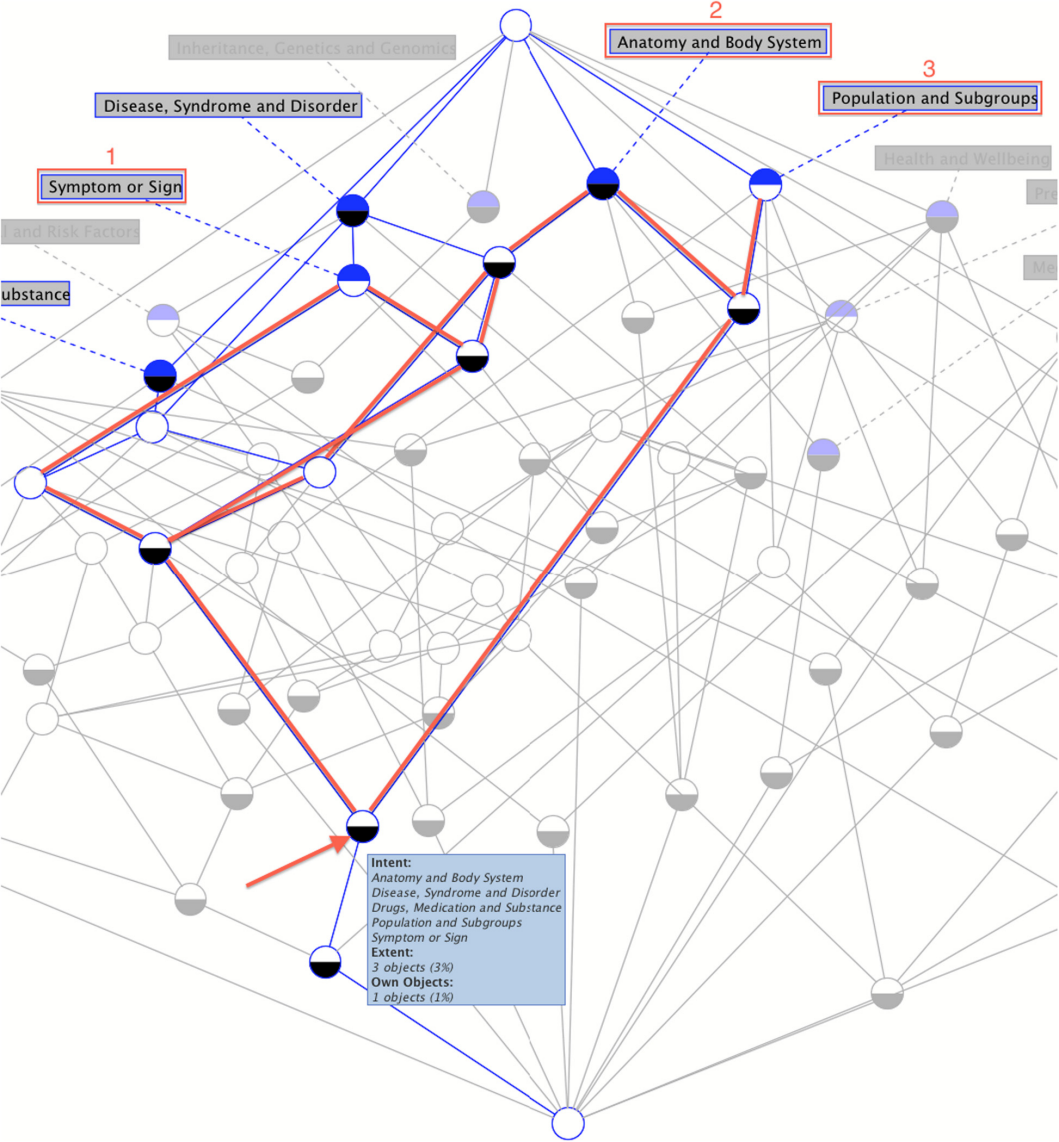
**The corresponding concept node in the lattice diagram for the selected categories in Figure **3**.** In the concept lattice, the node pointed by the arrow reflects three topics determined by the three selected categories as selected in Figure 3 (selecting additional categories such as “Disease, Syndrome and Disorder,” and “Drugs, Medication and Substance” does not change the resulting topics, since these are consequences determined by FCA).

The category-topic context and topic-question context dynamically drive the iCOACH prototype interface to allow the consumer multiple paths to quickly narrow down to relevant questions (Figure 3). Based on the category-topic context, a set of selected categories (leftmost column of Figure 3) dynamically generates a set of topics involving all the selected categories (middle column of Figure 3). Figure 4 illustrates the corresponding concept node in the concept lattice diagram. This correspondence also demonstrates the elimination of the need for an explicit label for such a composite concept node, because our strategy has avoided using menu-hierarchies generated from the concept lattice. The effect of category selection and the corresponding nodes in the concept lattice are shown in Additional file 1: Figures S1-S6. Based on the topic-question context, a set of topics drives the dynamic generation of a set of questions (rightmost column of Figure 3).

## Discussion

### Contribution and distinction from existing work

Multi-topic assignment is a challenging topic. When each question is assigned to one or several topics among nearly a hundred candidates, the probability of getting even a small fraction of tens of thousands of questions correct at random is virtually zero.

Our multi-topic assignment method combines semantic annotation using UMLS concepts (CUIs) and FCA and attained an example-based precision of 0.849, recall of 0.774, and *F*_1_ measure of 0.782, using a reference standard of 278 questions with manually assigned topics. Our approach is non-standard. It is not a typical machine learning approach since we do not start with a training set for supervised learning. Neither is it a completely unsupervised approach since we do take advantage of existing knowledge in NetWellness. Our approach is perhaps best characterized as a hybrid version using a combination of information retrieval techniques and FCA.

Organizing consumer health information according to a conjunctive structure determined by FCA allows content items to be located from multiple pathways. It achieves a better “organizational precision and recall,” in the sense that items, if found, are in their right place (precision) and an item is located at where it should be (recall).

Our prototype interface iCOACH overcomes one of the disadvantages of the existing body of work on FCA-based menu-design and organization [22-24]. One of the main challenges in such an approach is finding appropriate labeling for composite concept nodes, representing a conjunctive aggregation of attributes. For example, finding a concise and accurate label for a new category representing the intersection of “Anatomy and Body System,” “Disease, Syndrome and Disorder,” and “Inheritance, Genetics and Genomics” is difficult, if not impossible. iCOACH avoids this problem by allowing multiple (conjunctive) selection by a user to drill-down to the corresponding concept node in the hierarchy automatically generated using FCA without having to name the concept node. This removes the need to navigate nested menus hierarchies as well.

iCOACH is distinct from faceted search [25], where each facet represents an independent, disjoint dimension of information, typically consisting of a set of “values.” The basic requirements for faceted search involves the design of the facets, the classification of entities into the facets, and interfaces allowing the user to intuitively interact with and navigate to targeted entities. iCOACH shares the navigational exploration objective of faceted search, without the constraints typically imposed to facets. Entities in iCOACH are consumer health questions, which do not come with a naturally useful set of facets and values. Typical facet values such as dates when questions are posted, the names of the experts who answered the questions, are of limited value for information seeking by the consumer. The topics a question is tagged with could serve as the basis for faceted search. But because a basic premise of faceted search is value “mutual exclusivity” [26], it prevents the assignment of multiple topics to a single question. iCOACH overcomes such constraints by employing “chain-linked” formal contexts to achieve a general facet-like navigational exploration framework, without requiring strict conformation to the mutual exclusivity of the standard notion of “facet” [26].

iCOACH is a generalization of the conjunctive exploratory navigation interface CENI [8] which allows consumer health questions in NetWellness to be retrieved from multiple health topics. CENI uses the topic-question formal context to drive the conjunctive exploration of health questions, while iCOACH uses chain-linked category-topic and topic-question contexts (two tiers) to explore health questions.

Similar to CENI, iCOACH reused NetWellness’ existing health topics, which may not represent the best choices of potential health topics. In separate work [27], a semantic conjunctive exploratory navigation interface (SCENI) is developed to retrieve health questions using UMLS concepts as potential topics.

### Performance analysis

In this paper, we focused on evaluating the performance of categorizing questions into multiple topics. Using the combined FCA approach (both topic-subject and CUI-question contexts) achieved the best example-based recall and *F*_1_ measure (Table 7). For the top 10 topics ranked by the number of questions in the reference standard according to the original NetWellness assignment (Table 8), the topic “Pregnancy” achieved the best example-based recall and *F*_1_ measure using our combined FCA approach. The reason may be that although pregnancy-related questions often were very specific, but they were also related to other problems occurring during pregnancy. Note that the topic “Diet and Nutrition” achieved the lowest *F*_1_ measure for our combined FCA approach. The reason might be that this is a “vague” topic, and questions that should be assigned to this topic did not necessarily mention the key words “diet” or “nutrition” explicitly.

Performance of categorizing questions into multiple topics is affected by a number of factors including the key CUIs selection algorithm, the quality of the topics to be assigned, and the quality of the questions themselves. To improve the performance of the basic step of tagging a question by relevant CUIs, we performed manual error review which indicated potential for further improvement. For example, two questions (Q1 and Q2) both asking about strong smell have the same key CUI-set {“C0442821: Strong”, “C0037361: Smell” }, which were correctly identified. However, the CUI-set did not completely determine the topic in this case, since Q1 asked about strong smell in diaper and assigned the topic of “Children’s Health,” while Q2 was more about a smell disorder related to the topic of “Ear, Nose, and Throat Disorders.” Further CUI-set selection including those tagged for answers may help address this issue. The quality of NetWellness contents naturally affects performance. Some NetWellness topics are too general. Some NetWellness questions are too short to be informative, and some too long. These represent the source of typical false negatives or false positives. An area of immediate opportunity is to redesign a collection of consumer health topics by refining and expanding the existing set of 99 topics.

### Limitation

Our evaluation has been focused the on the multi-topic assignment problem. The iCOACH interface has not been directly evaluated by external users. However, its simplification CENI [8] was evaluated through a crowdsourcing search-interface comparative study with Amazon Mechanical Turk, which showed the effectiveness of the conjunctive organization and exploration of health questions by multiple topics.

## Conclusions

Enhancing the recall of multi-topic assignment without sacrificing precision is a prerequisite for achieving the benefits of navigational exploration. Our new multi-topic assignment method, combining term-strength, FCA, and information retrieval techniques, significantly improved recall and performed well according to established metrics. iCOACH provides an environment for organizing about 60,000 existing questions in NetWellness for navigational exploration. By organizing consumer health information sources such as NetWellness at the levels of categories, topics, and questions (contents), multiple entry points are offered for the consumer to explore information of interest, even though the precise terms for searching such information may be non-trivial or difficult to formulate.

## Appendix

We present a brief example to help illustrate the differences between example-based evaluation measures and traditional evaluation measures. Table 9 is an example consisting of four questions *x*_1_,*x*_2_,*x*_3_,*x*_4_ and their topic assignments in the reference standard *Y*_*i*_ and predicted topic assignments *Z*_*i*_.

**Table 9:** **Questions *x***_**1**_**, *x***_**2**_**, *x***_**3**_**, *x***_**4**_** and their topic assignments in the reference standard *Y***_***i***_** and predicted topic assignments *Z***_***i***_

***x***_***i***_	***Y***_***i***_** (Reference standard)**	***Z***_***i***_** (Predicted assignment)**
*x*_1_	{a, b}	{a, b}
*x*_2_	{a, c}	{a, c}
*x*_3_	{d}	{d}
*x*_4_	{a, b, c, d, e, f, g, h, k, l}	{a, s}

Table 10 shows how the example-based precision (*P*), recall (*R*), and *F*_1_ measure (*F*_1_) are computed using Eq. 1, Eq. 2 and Eq. 3 in Subsection “Evaluation metrics.” A precision of 0.875, recall of 0.775, and *F*_1_ measure of 0.792 are obtained as indicated in the last row.

**Table 10:** Evaluation based on example-based metrics (macro-average on the example level)

***x***_***i***_	***|Y***_***i***_***|***	***|Z***_***i***_***|***	***|Y***_***i***_*** ∩ Z***_***i***_***|***	$\frac{\left|Y_{i}\cap Z_{i}\right|}{\left|Z_{i}\right|}$	$\frac{\left|Y_{i}\cap Z_{i}\right|}{\left|Y_{i}\right|}$	$\frac{2|Y_{i}\cap Z_{i}|}{\left|Z_{i}|+|Y_{i}\right|}$
*x*_1_	2	2	2	1	1	1
*x*_2_	2	2	2	1	1	1
*x*_3_	1	1	1	1	1	1
*x*_4_	10	2	1	0.5	0.1	0.167
Macro-average				*P *= **0.875**	*R *= **0.775**	*F*_1 _= **0.792**

The following steps show how the traditional evaluation metrics are calculated resulting a lower recall (0.438) and *F*_1_ value (0.584), where *TP*, *FN*, and *FP* represent numbers of true positives, false negatives, and false positives, respectively.

$$\begin{array}{lll} & \text{TP}=\sum_{i=1}^{4}|Y_{i}\cap Z_{i}|=2+2+1+2=7\; & \;\\ & \text{FN}=\sum_{i=1}^{4}(|Y_{i}|-|Y_{i}\cap Z_{i}|)=0+0+0+9=9\; & \;\\ & \text{FP}=\sum_{i=1}^{4}(|Z_{i}|-|Y_{i}\cap Z_{i}|)=0+0+0+1=1\; & \;\\ & P=\text{TP}/(\text{TP}+\text{FP})=7/8=\text{0.875}\; & \;\\ & R=\text{TP}/(\text{TP}+\text{FN})=7/16=\text{0.438}\; & \;\\ & F_{1}=2\times P\times R/(P+R)=\text{0.584}\; & \; \end{array}$$

As illustrated above, using traditional methods allows the performance on a few questions (such as *x*_4_) with an unusually large number of labels to dominate the values of evaluation metrics (which is undesirable), while example-based metrics have been designed to avoid undue influence of such questions.

## Abbreviations

CQA: Community question answering; CUI: Concept unique identifier; FCA: Formal concept analysis; iCOACH: Concept-guided automatic organization of consumer health information.

## Competing interests

The authors declare that they have no competing interests.

## Authors’ contributions

LC and GQZ conceptualized and designed the iCOACH approach. LC implemented the algorithms and interface with input from GQZ. LC, GQZ, and RX performed the evaluation. LC, GQZ lead the writing of the paper, with critical reviews by ZL and RX. SW and KS provided domain expertise and assisted the evaluation. All authors read and approved the final manuscript.

## Pre-publication history

The pre-publication history for this paper can be accessed here:

http://www.biomedcentral.com/1472-6947/14/63/prepub

## Supplementary Material

Additional file 1 **Supplement Materials.** The file include supplement tables and figures as follows: Figure S1. User selects the “Symptom or Sign” category in the iCOACH prototype interface. Relevant Health Topics are displayed automatically. Figure S2. After selecting a category “Symptom or Sign,” the corresponding node in the concept lattice (with 71 concept nodes) of the category-topic context is indicated. Even though the category “Disease, Syndrome and Disorder” is not selected by the user, it is an “implicant” of the selected category due to the logic of FCA. Figure S3. User selects the second category, “Anatomy and Body System,” in the iCOACH prototype interface. The corresponding Health Topics relevant to both categories are automatically displayed (this is an updated list). Figure S4. The arrow in this figure points to the corresponding node in the diagram of the lattice after selecting the indicated categories in Figure S3. Again, even though the category “Drugs, Medication and Substance” was not selected by the user, it is a logical consequence of the selected categories, inferred by FCA. Figure S5. Finally, user selects the third category, “Population and Subgroups,” in the iCOACH prototype interface. The corresponding Health Topics now narrows down to only three that are relevant to all the selected categories. Figure S6. The arrow in this figure points to the corresponding node in the diagram of the lattice after selecting the indicated categories in Figure S5. Note that as more categories are selected, the corresponding concept node moves further down in the lattice hierarchy, covering fewer health topics. This demonstrates the duality in FCA with respect to attributes (in this case Categories) and their corresponding objects (in this case Health Topics): more attributes serve to narrow down to fewer objects with all the relevant attributes. Click here for file
