# Supplementary material for: Multi-topic assignment for exploratory navigation of consumer health information in NetWellness using formal concept analysis
Source: BMC Med Inform Decis Mak. 2014 Aug 3;14:63. doi: 10.1186/1472-6947-14-63 (PMC4131492; doi:10.1186/1472-6947-14-63)
Supplement: Additional file 1 — Supplement Materials. The file include supplement tables and figures as follows: Figure S1. User selects the “Symptom or Sign” category in the iCOACH prototype interface. Relevant Health Topics are displayed automatically. Figure S2. After selecting a category “Symptom or Sign,” the corresponding node in the concept lattice (with 71 concept nodes) of the category-topic context is indicated. Even though the category “Disease, Syndrome and Disorder” is not selected by the user, it is an “implicant” of the selected category due to the logic of FCA. Figure S3. User selects the second category, “Anatomy and Body System,” in the iCOACH prototype interface. The corresponding Health Topics relevant to both categories are automatically displayed (this is an updated list). Figure S4. The arrow in this figure points to the corresponding node in the diagram of the lattice after selecting the indicated categories in Figure S3. Again, even though the category “Drugs, Medication and Substance” was not selected by the user, it is a logical consequence of the selected categories, inferred by FCA. Figure S5. Finally, user selects the third category, “Population and Subgroups,” in the iCOACH prototype interface. The corresponding Health Topics now narrows down to only three that are relevant to all the selected categories. Figure S6. The arrow in this figure points to the corresponding node in the diagram of the lattice after selecting the indicated categories in Figure S5. Note that as more categories are selected, the corresponding concept node moves further down in the lattice hierarchy, covering fewer health topics. This demonstrates the duality in FCA with respect to attributes (in this case Categories) and their corresponding objects (in this case Health Topics): more attributes serve to narrow down to fewer objects with all the relevant attributes. [file 1472-6947-14-63-S1.pdf]

Supplement Materials

Table S1. The topic CUI-set for "Pulmonary Fibrosis" (27CUIs)

CUI	Concept Name
C0034069	Pulmonary Fibrosis
C0264530	Perialveolar fibrosis of lung
C1800706	Usual interstitial pneumonitis
C0865849	Diffuse Pulmonary Fibrosis
C0175999	Post-inflammatory pulmonary fibrosis
C0311227	Aluminosis of lung
C0340183	Mica pneumoconiosis
C0270169	Interstitial pulmonary fibrosis of prematurity
C0264531	Peribronchial fibrosis of lung
C0264437	Bauxite fibrosis of lung
C0340127	Localized pulmonary fibrosis
C0041336	Tuberculous fibrosis of lung
C0264439	Graphite fibrosis of lung
C0340126	Fibrosis of lung following radiation
C0264512	Rheumatoid fibrosing alveolitis
C2350622	Radiation Fibrosis
C1320687	Post-radiotherapy pulmonary fibrosis
C0206061	Pneumonia, Interstitial
C0264529	Massive fibrosis of lung
C0264528	Confluent fibrosis of lung
C0347840	O/E - fibrosis of lung present
C0264507	Prolonged pulmonary alveolitis
C0264506	Simple pulmonary alveolitis
C0340191	Subacute silicosis
C0264527	Atrophic fibrosis of lung
C0264526	Chronic fibrosis of lung
C0085786	Hamman-Rich syndrome

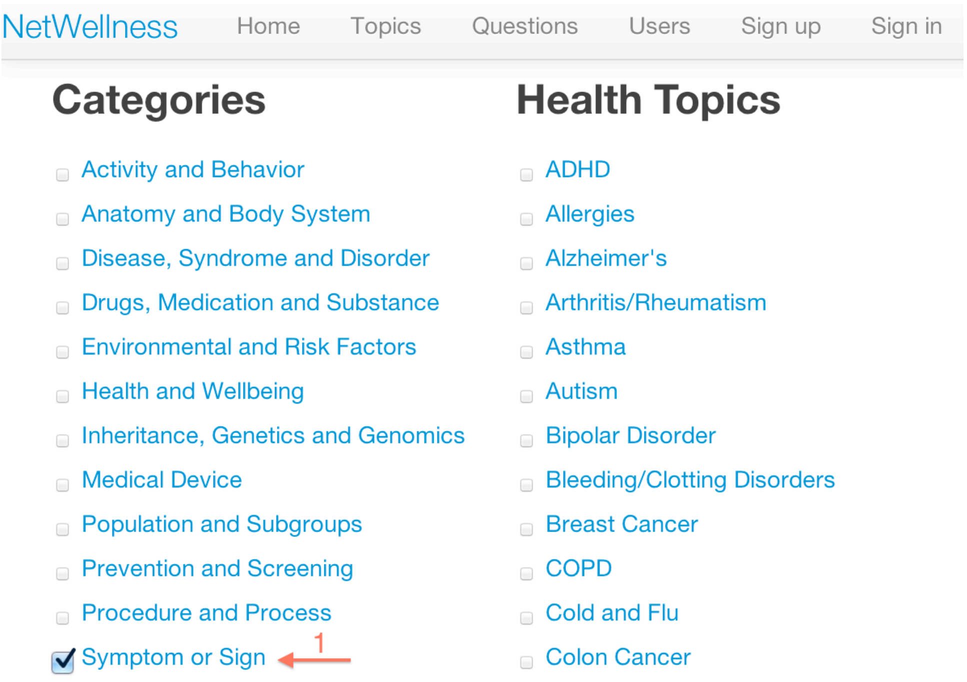

Figure S1. User selects the “Symptom or Sign” category in the iCOACH prototype interface. Relevant Health Topics are displayed automatically.

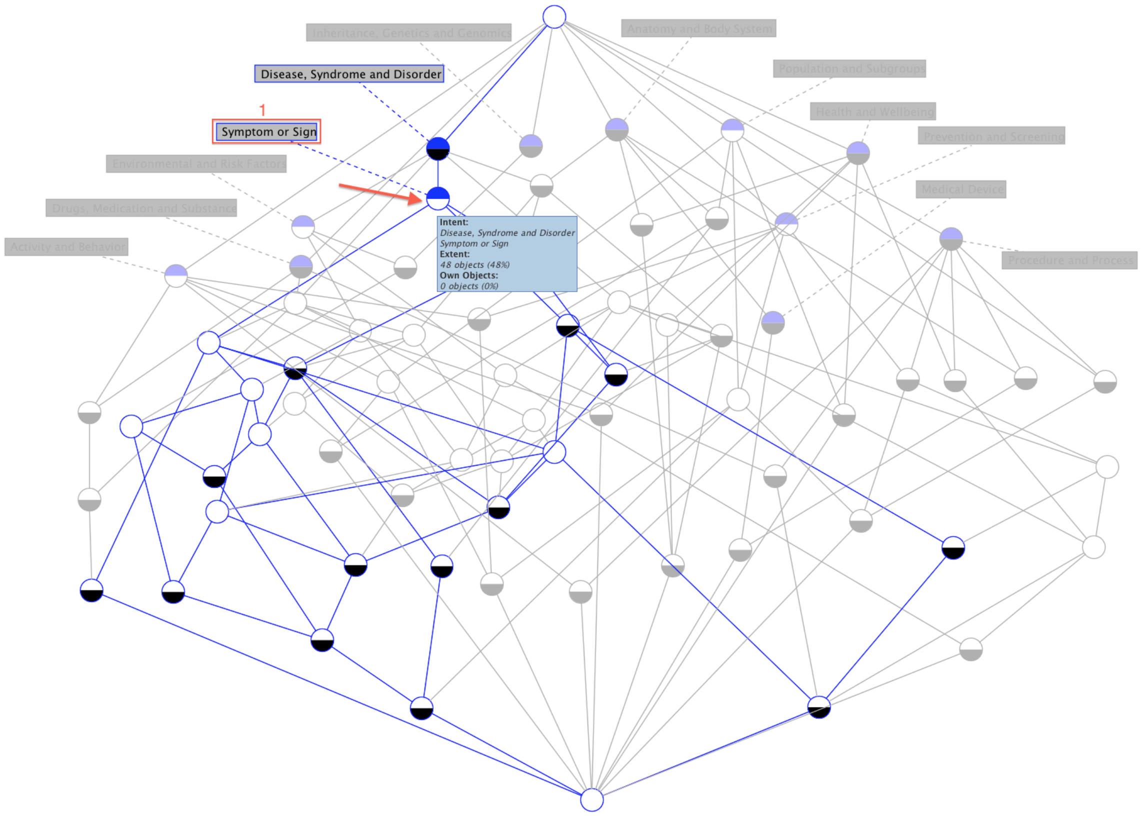

Figure S2. After selecting a category “Symptom or Sign,” the corresponding node in the concept lattice (with 71 concept nodes) of the category-topic context is indicated. Even though the category “Disease, Syndrome and Disorder” is not selected by the user, it is an “implicant” of the selected category due to the logic of FCA.

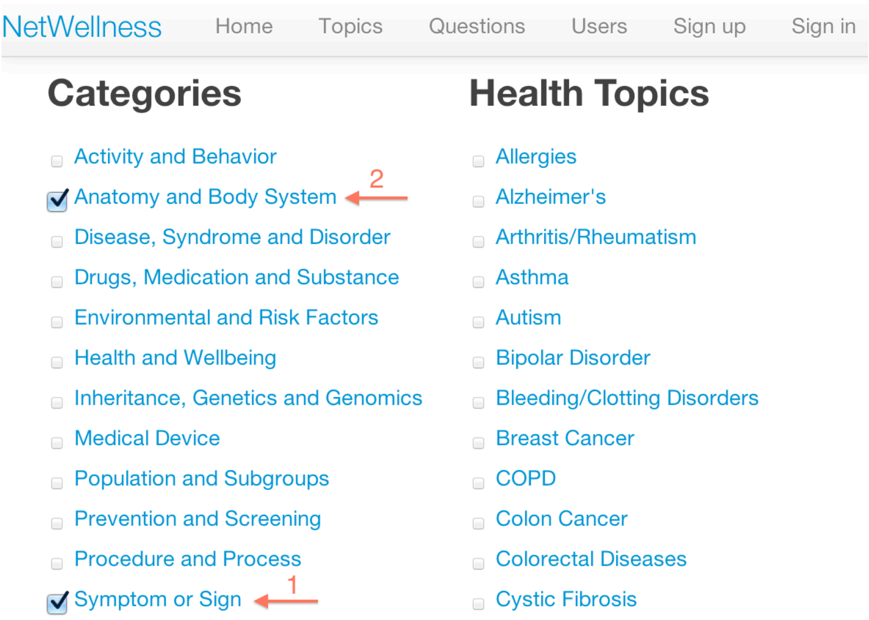

Figure S3. User selects the second category, “Anatomy and Body System,” in the iCOACH prototype interface. The corresponding Health Topics relevant to both categories are automatically displayed (this is an updated list).

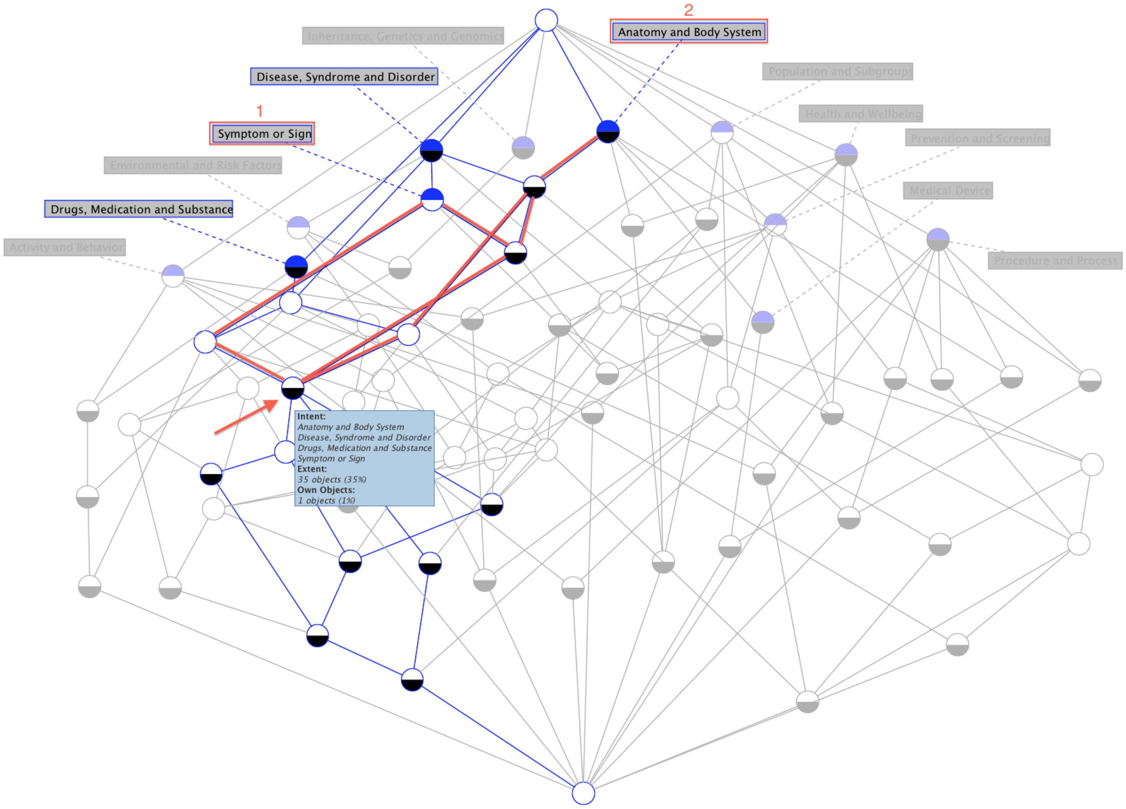

Figure S4. The arrow in this figure points to the corresponding node in the diagram of the lattice after selecting the indicated categories in Figure S3. Again, even though the category “Drugs, Medication and Substance” was not selected by the user, it is a logical consequence of the selected categories, inferred by FCA.

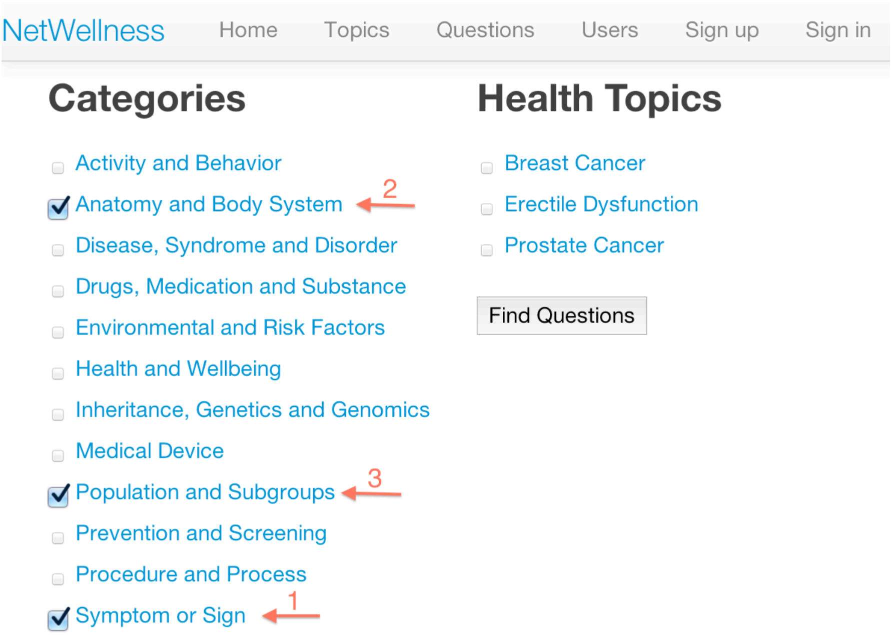

Figure S5. Finally, user selects the third category, “Population and Subgroups,” in the iCOACH prototype interface. The corresponding Health Topics now narrows down to only three that are relevant to all the selected categories.

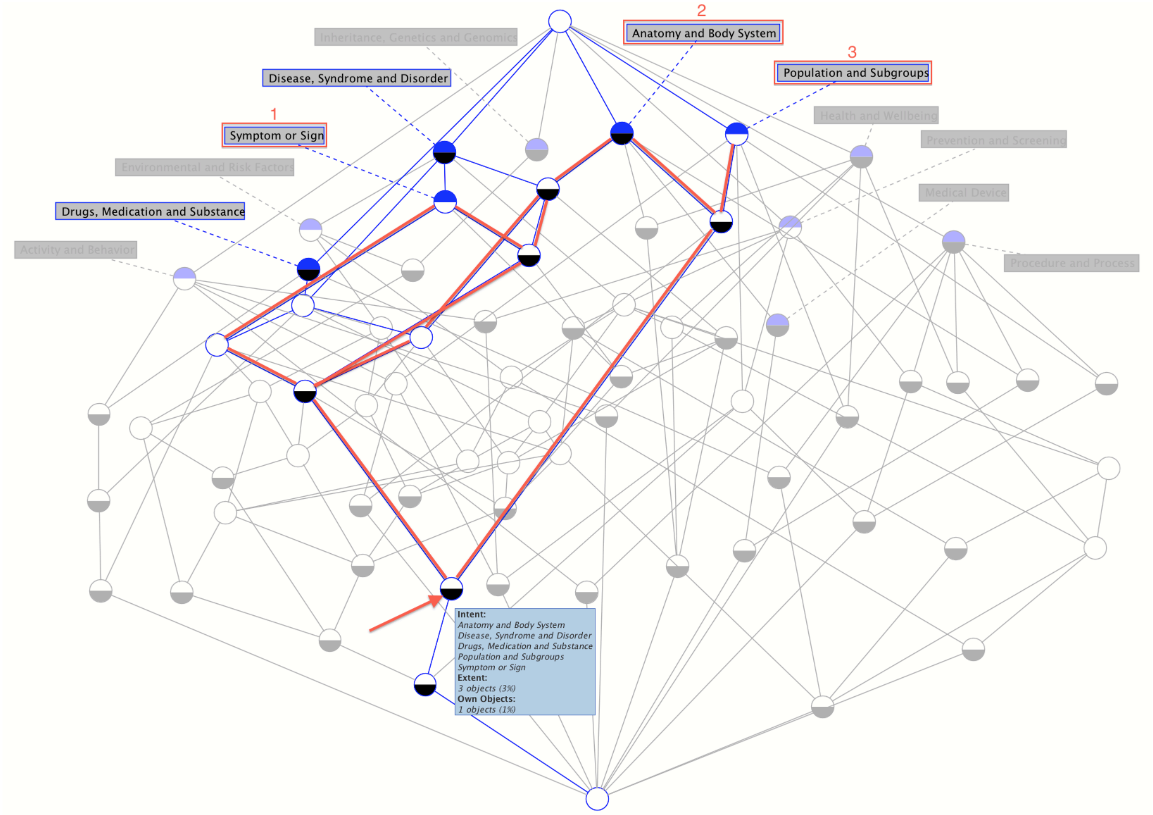

Figure S6. The arrow in this figure points to the corresponding node in the diagram of the lattice after selecting the indicated categories in Figure S5. Note that as more categories are selected, the corresponding concept node moves further down in the lattice hierarchy, covering fewer health topics. This demonstrates the duality in FCA with respect to attributes (in this case Categories) and their corresponding objects (in this case Health Topics): more attributes serve to narrow down to fewer objects with all the relevant attributes.
